# Challenges Faced by Parents of Children With Selective Mutism in Accessing Medical Care Services

**DOI:** 10.7759/cureus.71304

**Published:** 2024-10-12

**Authors:** Tomohisa Yamanaka, Kengo Yuruki, Taro Okano, Masahiko Inoue

**Affiliations:** 1 Department of Doctoral Course, Graduate School of Medical Sciences, Tottori University, Yonago, JPN; 2 Student Accessibility Office, Head Office for Education and Student Support, Shimane University, Matsue, JPN; 3 Department of Assessment, Tottori Prefecture Welfare Counseling Center, Tottori, JPN; 4 Faculty of Human Studies, Seisen University, Hikone, JPN; 5 Department of Clinical Psychology, Graduate School of Medical Sciences, Tottori University, Yonago, JPN

**Keywords:** barriers to healthcare, healthcare survey, parent, qualitative analysis, selective mutism

## Abstract

Background

Children with selective mutism (SM) typically exhibit SM symptoms in educational settings, which has led to extensive research on SM in such environments. However, SM symptoms also manifest in medical settings, where parents experience various challenges when seeking healthcare for their child with SM. Nevertheless, there is a lack of research on the specific challenges parents face when seeking medical care for their child with SM. In this study, we investigated the challenges faced by parents of children with SM when seeking healthcare services and explored strategies to reduce these challenges.

Methodology

In this study, we conducted an online questionnaire survey with 31 parents (mean age: 41.7 years) of children diagnosed with SM. Participants responded to open-ended questions about the difficulties they faced when seeking medical care for their child. The open-ended responses were qualitatively analyzed using the KJ method.

Results

Parents of children with SM reported the most challenges accessing medical services in pediatrics and dentistry. The analysis identified the following three main challenges parents face when seeking medical care for their child with SM: “Journey to Receiving Treatment,” “Physicians’ Inability to Accurately Capture the Child’s Condition,” and “Healthcare Providers’ Responses.”

Conclusions

These findings highlight the numerous challenges faced by parents of children with SM when seeking medical services for their child. The challenges faced by parents when bringing their child with SM to medical facilities may be alleviated through the implementation of telemedicine, the development of mobile health applications, multidisciplinary collaboration, and the introduction of SM-specific training programs for healthcare professionals.

## Introduction

Selective mutism (SM) is an anxiety disorder characterized by a consistent failure to speak in specific social situations, such as kindergarten or school, despite the ability to speak in other settings [1]. Genetic and temperamental factors have been suggested to be involved in the onset of SM. Stein et al. linked the *CNTNAP2* gene to SM, suggesting a genetic influence [2], while Gensthaler et al. identified behavioral inhibition as a key temperamental factor contributing to SM [3]. The prevalence of SM is estimated to range from 0.18% to 1.90% [4]. It typically manifests around age 4.6 (range: 2.9 to 5.2 years) and can follow various courses, with some cases resolving early and others persisting into adulthood [5,6]. Recent studies have provided new insights into the prognosis of individuals who experience SM, showing that even when these individuals become able to speak in any situation, they tend to have higher interpersonal anxiety, lower communication skills, and reduced self-esteem compared to those without SM [7].

SM typically occurs in school or kindergarten settings, prompting numerous studies in educational environments [8-10]. However, as SM is characterized by a consistent failure to speak in specific social situations, these symptoms can also arise in medical settings. When children with SM experience anxiety in these environments, they may hide their symptoms from both healthcare providers and family members [11]. Additionally, even when seeking medical care, a lack of understanding from healthcare providers can hinder effective support. Hisada and Hamada reported that some individuals with SM encountered physicians who expressed disbelief, with one patient being told, “Usually, it becomes too uncomfortable, and people end up talking” [12].

A study by Keville et al. found that parents of children with co-occurring SM and autism spectrum disorder (ASD) face five major challenges, including “the diagnostic journey” [13]. This challenge was composed of the following two sub-themes: “Navigating the minefield” and “A lengthy process: Hindered development and hidden abilities.” In “Navigating the minefield,” parents feared delayed diagnoses would hinder their children’s development and worsen their mental health. They also worried that a prolonged diagnostic process would delay access to necessary support, negatively impacting their children’s lives and education. In “A lengthy process,” parents noted that the healthcare and educational systems often hinder diagnosis and support, forcing them to arrange diagnoses and treatments independently.

These observations suggest that parents of children with SM may encounter various challenges when seeking medical care. However, the specific challenges parents face when bringing a child with SM to a medical facility have not been thoroughly investigated. Understanding these challenges is crucial, as addressing them could improve access to healthcare for children with SM, reduce the burden on parents, and contribute to the overall health and well-being of both the children with SM and their families. This study qualitatively analyzed open-ended responses obtained from an online questionnaire to explore the challenges parents of children with SM encounter when seeking medical care. This study aims to identify these challenges and examine strategies for alleviating the challenges parents face when bringing their children with SM to medical facilities.

A summary of this study was presented as an online poster at the 64th Annual Meeting of the Japanese Society for Child and Adolescent Psychiatry, held from November 14 to 16, 2023.

## Materials and methods

Study design

Given the low prevalence of SM in the population [4], conducting large-scale sampling within a specific region presents significant challenges. Online surveys allow the collection of large, geographically diverse samples and facilitate targeting specific groups [14]. Additionally, the anonymity provided by online surveys encourages more candid responses to sensitive issues, reducing social desirability bias [15]. Based on these considerations, the implementation of an online survey was deemed instrumental in achieving the objectives of this study.

Open-ended responses from parents of children with SM were analyzed using the KJ method [16], a prominent qualitative analysis technique. The KJ method was chosen for its systematic approach, which enabled the study to uncover critical insights into the medical care challenges faced by parents of children with SM. The KJ method organizes qualitative data by identifying meaningful insights through data synthesis [16,17]. This method involves extracting meaningful segments from participants’ responses, categorizing them based on similarities, and constructing a hierarchical structure to identify key themes.

Study participants

In this study, convenience sampling was used. This method involved recruiting participants online, where individuals meeting specific criteria voluntarily participated in the survey. We recruited participants by contacting parents of children with SM through support organizations for SM and websites dedicated to participant recruitment for this study. The study population comprised parents of children diagnosed with SM. Given the high prevalence of comorbid neurodevelopmental and anxiety disorders in children with SM, we also included parents of children with these comorbidities [18,19]. Therefore, the inclusion criteria were based on the following five conditions: (1) families with children aged 4-17 years who had a diagnosis of SM. (2) Families with children aged 4-17 years who were suspected to have SM. This age range was selected because SM typically manifests during early childhood. The upper limit of 17 years was chosen to focus on children and adolescents, as developmental differences arise significantly after 18 years of age. (3) Respondents aged 20 years or older. (4) Individuals capable of responding via survey form. (5) Individuals who owned a device capable of accessing the survey form. Exclusion criteria were as follows: (1) Individuals deemed unsuitable for the study by the researchers for any reason.

Study questionnaire

The questionnaire included items on demographic variables such as the participant’s age and gender, the child’s age, the presence of an SM diagnosis, and any comorbid diagnoses. It also contained open-ended questions, such as “What difficulties do you experience when seeking medical care for your child?” Respondents were instructed to describe the medical departments where these difficulties occurred, with no limit on the number of departments they could mention. The details of the responses to the questionnaire are shown in Appendix 1.

Data analysis

The analysis procedure, based on the KJ method, followed these steps: first, meaningful segments were extracted from the open-ended responses, ensuring that each segment represented only one meaning. If a response contained multiple meanings, it was divided into separate segments. In the next step, all extracted segments were reviewed multiple times. Segments with similar meanings were grouped into a first-level category, with an appropriate group name assigned. Segments that did not share similar meanings were not forced into a category. Next, the names of the existing categories and any remaining segments were reviewed, grouping those with similar meanings or names into the second-level category. This process continued with the previously formed categories and ungrouped segments, forming higher-order categories by grouping similar characteristics. This categorization was repeated until the fourth level, where no further similarities could be identified. Finally, four clinical psychology experts reviewed and finalized the categories, assessing the appropriateness of the category names and classifications.

Ethical considerations

This study was approved by the Ethical Review Committee of Tottori University School of Medicine (approval number: 21A174). The study was conducted in accordance with the ethical standards of the 1964 Declaration of Helsinki and its subsequent amendments.

## Results

Demographic information related to parents, children, comorbidities, and medical departments with reported difficulties is included in Table 1.

**Table 1:** Demographic information on parents, children, comorbidities, and medical departments with reported difficulties. ASD: autism spectrum disorder; ADHD: attention-deficit/hyperactivity disorder; ID: intellectual disability; SepAD: separation anxiety disorder; SM: selective mutism; SocAD: social anxiety disorder

Variable	Summary
Parents’ mean age	41.77 (SD = 4.54, range = 32–48)
Parents’ gender	Female = 31 (100%)
Children’s mean age	8.96 (SD = 3.14, range = 4–16)
Children’s gender	Female = 23 (74.19%), Male = 8 (25.80%)
Children’s mean age at SM diagnosis	5.96 (SD = 2.30, range = 3–13)
Children’s comorbidities	
ASD	21 (67.7%)
ADHD	3 (9.67%)
SocAD	3 (9.67%)
ID	2 (6.45%)
SepAD	1 (3.22%)
Medical departments with reported difficulties	
Pediatrics	17 (53.12%)
Dentistry	17 (53.12%)
Otolaryngology	9 (28.12%)
Ophthalmology	8 (25.00%)
Psychiatry	6 (18.75%)
Dermatology	4 (12.5%)
Internal Medicine	3 (9.37%)
Orthopedics	1 (3.12%)

Participants’ demographic information

Participants were recruited through convenience sampling, and 32 parents of children diagnosed with SM agreed to participate, reflecting the number of respondents willing to participate during the recruitment period. One parent was excluded from the analysis because their child did not meet the inclusion criteria, resulting in a final sample of 31 parents, all of whom were female. The mean age of the parents was 41.77 years (SD = 4.54 years, range = 32-48).

Age and comorbidities in children with selective mutism

The mean age of the children was 8.96 years (SD = 3.14, range 4-16 years). Of the 31 children with SM, 23 were female and eight were male. The average age at which they were diagnosed with SM was 5.96 years (SD = 2.30 years, range = 3-13). Ten children had an SM diagnosis only, while 21 children had comorbidities (ASD, n = 21; attention-deficit/hyperactivity disorder, n = 3; social anxiety disorder, n = 3; intellectual disability, n = 2; separation anxiety disorder, n = 1).

Departments where parents of children with selective mutism encountered difficulties in accessing medical services

The medical departments where parents of children with SM most frequently reported difficulties were pediatrics and dentistry, with 17 reports each. This was followed by otolaryngology with nine reports, ophthalmology with eight, psychiatry with six, dermatology with four, internal medicine with three, and orthopedics with one.

Analysis of the open-ended question

**Figure 1:**
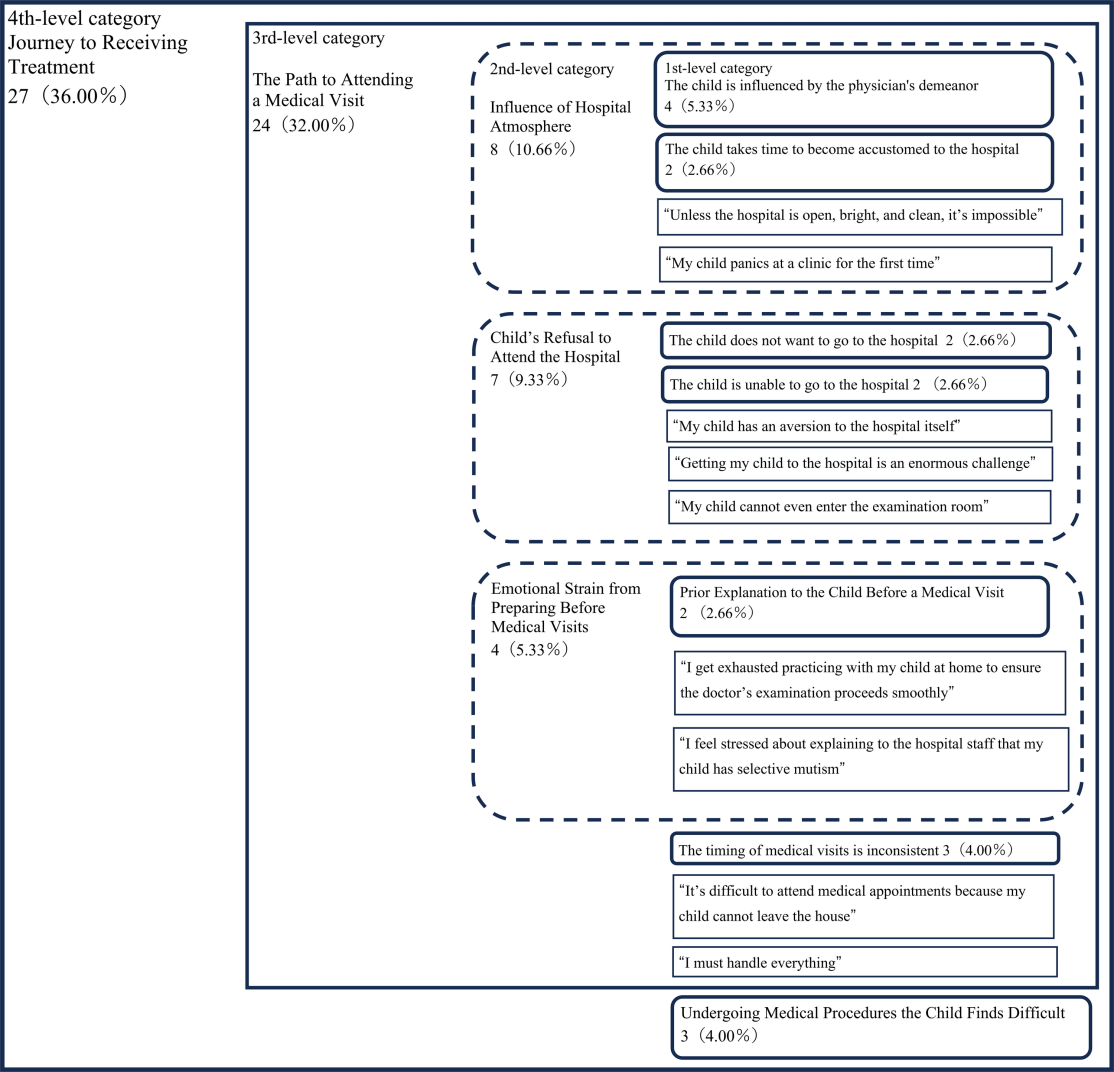
KJ method analysis results for the category “Journey to Receiving Treatment.” The fourth-level category represents the broadest theme, while the third-level category breaks this down into more specific areas of focus. Each second-level category then further narrows these areas into specific themes, and the first-level categories represent sub-themes or individual aspects related to these themes. The numbers represent the number of segments that mentioned the content belonging to each category, and the numbers in parentheses indicate the percentage of the total number of segments within the group.

**Figure 2:**
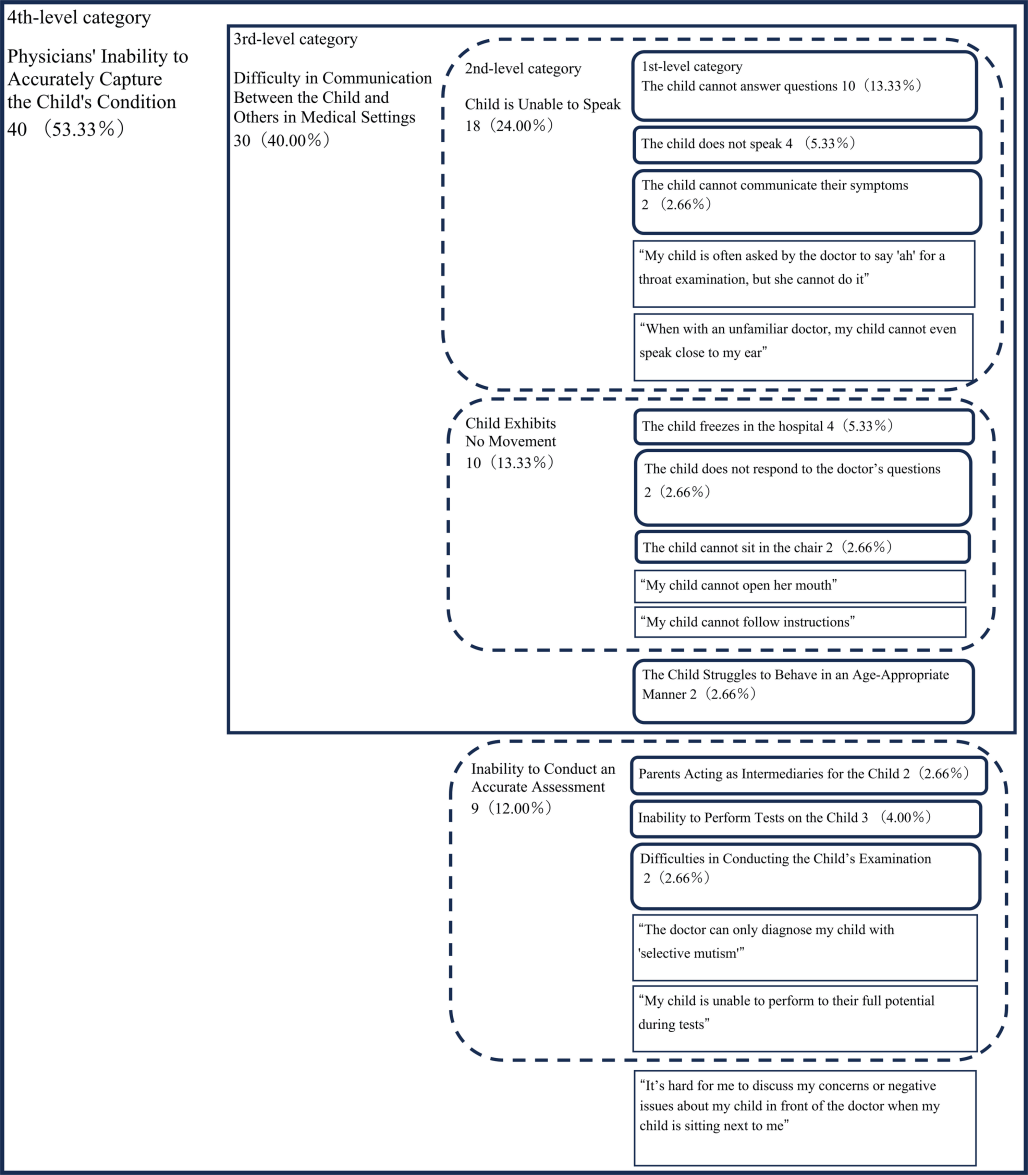
KJ method analysis results for the category “Physicians; Inability to Accurately Capture the Child’s Condition.” The numbers represent the number of segments that mentioned the content belonging to each category, and the numbers in parentheses indicate the percentage of the total number of segments within the group.

**Figure 3:**
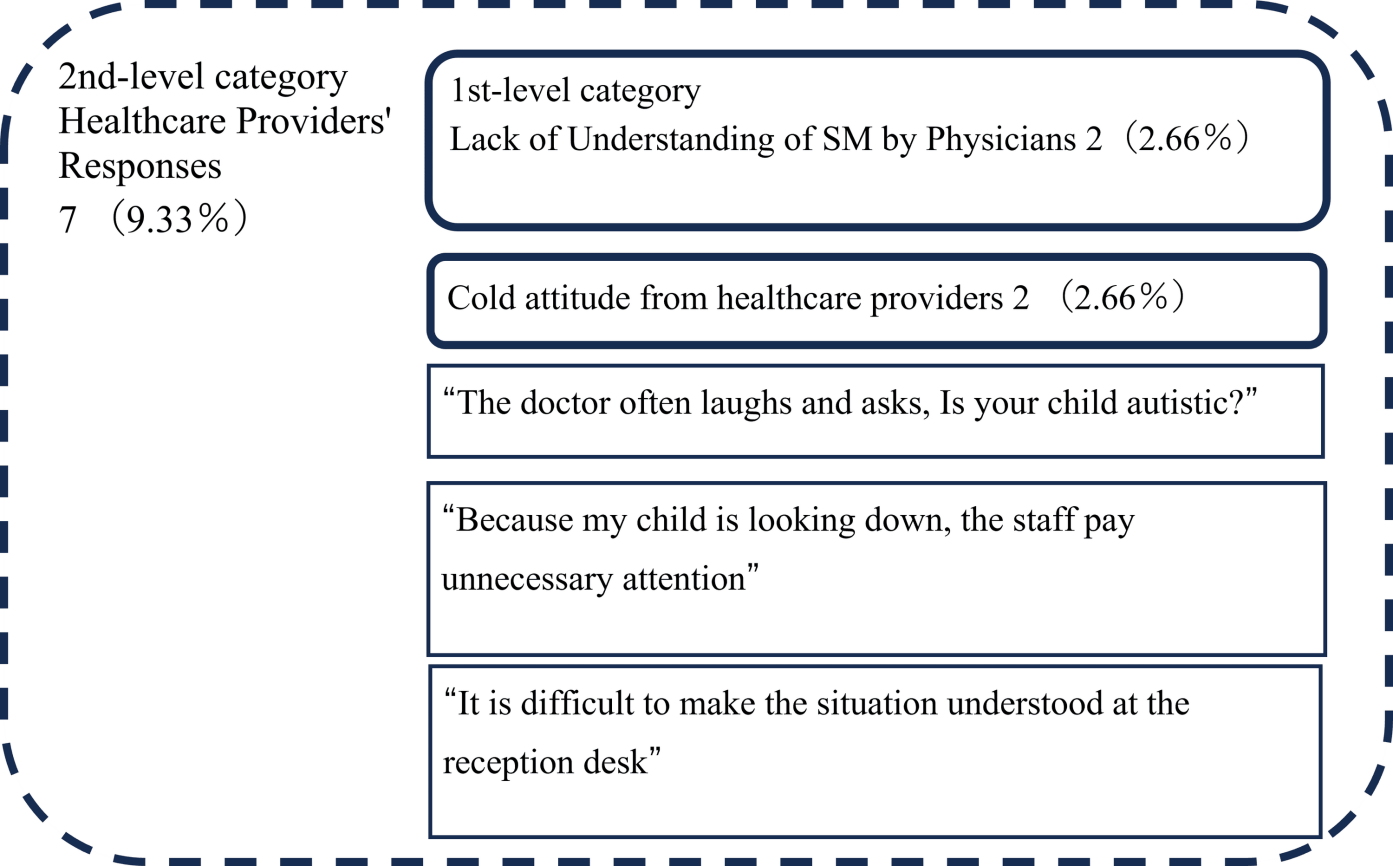
KJ method analysis results for the category “Healthcare Providers’ Responses.” The numbers represent the number of segments that mentioned the content belonging to each category, and the numbers in parentheses indicate the percentage of the total number of segments within the group.

The analysis using the KJ method resulted in the identification of 75 segments, which were organized into a total of 30 categories. There were 19 first-level categories, seven second-level categories, two third-level categories, and two fourth-level categories. Ultimately, these were categorized into the following three dimensions: “Journey to Receiving Treatment” (Figure 1), “Physicians’ Inability to Accurately Capture the Child’s Condition” (Figure 2), and “Healthcare Providers’ Responses” (Figure 3).

The category “Journey to Receiving Treatment” included categories such as the third-level category “The Path to Attending a Medical Visit” and the first-level category “Undergoing Medical Procedures the Child Finds Difficult”. The third-level category “The Path to Attending a Medical Visit” included second-level categories such as “Influence of Hospital Atmosphere,” “Child’s Refusal to Attend the Hospital,” and “Emotional Strain from Preparing Before Medical Visits” and the first-level category “The timing of medical visits is inconsistent.”

The category “Physicians’ Inability to Accurately Capture the Child’s Condition” included categories such as the third-level category “Difficulty in Communication Between the Child and Others in Medical Settings” and the second-level category “Inability to Conduct an Accurate Assessment,” as well as segments such as “It’s hard for me to discuss my concerns or negative issues about my child in front of the doctor when my child is sitting next to me.” The third-level category “Difficulty in Communication Between the Child and Others in Medical Settings” included the second-level categories such as “Child is Unable to Speak,” “Child Exhibits No Movement,” and the first-level categories “The Child Struggles to Behave in an Age-Appropriate Manner.” The second-level category “Inability to Conduct an Accurate Assessment” encompassed first-level categories such as “Parents Acting as Intermediaries for the Child,” “Inability to Perform Tests on the Child,” and “Difficulties in Conducting the Child’s Examination.” It also included segments such as “My child is unable to perform to their full potential during tests.”

The category “Healthcare Providers’ Responses” includes first-level categories such as “Lack of Understanding of SM by Physicians” and “Cold attitude from healthcare providers,” along with segments such as “The doctor often laughs and asks, ‘Is your child autistic’” and “It is difficult to make the situation understood at the reception desk.”

## Discussion

This study aimed to identify the challenges faced by parents of children with SM when accessing medical care and to explore the direction of future support for these parents. A qualitative analysis using the KJ method was conducted on open-ended responses obtained from an online questionnaire, revealing three dimensions: “Journey to Receiving Treatment,” “Physicians’ Inability to Accurately Capture the Child’s Condition," and “Healthcare Providers’ Responses.” Based on these findings, we discuss potential support strategies to alleviate the challenges parents encounter when seeking medical care for children with SM.

The findings in the category “Journey to Receiving Treatment” were consistent with a previous study on “The Diagnostic Journey”[13]. This category includes challenges such as children being afraid to visit the hospital, refusing to leave the house, or resisting entering the examination room. These challenges include the difficulty parents face in persuading or explaining to their child the need for a medical visit beforehand. Furthermore, it was suggested that even when parents manage to bring their child to the hospital, challenges may still arise if the physician is perceived as intimidating or the hospital environment feels unwelcoming, potentially causing the child to refuse the examination. One potential solution to alleviate or resolve the challenges faced by parents of children with SM when accessing medical services is the use of telemedicine. Recent studies have indicated that remote interventions for anxiety and related symptoms can be as effective as in-person therapy [20]. Additionally, for children with SM who refuse to visit support facilities, the usefulness of interventions using telemedicine has been reported [21]. Another potential solution is the implementation of psychological preparation. In the treatment of children with ASD, psychological preparation has been shown to be effective in reducing their fear during medical examinations and treatments. This approach involves medical staff carefully explaining examination procedures and treatment steps to children with ASD using illustrations or photographs to alleviate their fear [22]. Therefore, offering on-demand introductions to specific procedures and locations within medical facilities could potentially reduce the anxiety children with SM experience when visiting these settings.

The category “Physicians’ Inability to Accurately Capture the Child’s Condition” clearly indicates that SM symptoms are prominently present even in medical settings. As a result of their child’s SM symptoms, parents struggle to accurately convey their child’s condition to the physician, leading to significant challenges. One potential solution to alleviate the challenges faced by parents of children with SM is to implement strategies aimed at reducing the child’s anxiety during medical visits. For instance, using tablets or communication cards could facilitate communication for children with SM [23,24]. Another potential solution is the introduction of mobile health technology. The second-level category “Inability to Conduct an Accurate Assessment,” which is part of “Physicians’ Inability to Accurately Capture the Child’s Condition,” includes first-level categories such as “Parents Acting as Intermediaries for the Child,” “Inability to Perform Tests on the Child,” and “Difficulties in Conducting Examinations.” Information provided by parents may not always accurately reflect the child’s subjective experiences, complicating the physician’s ability to develop precise diagnoses and treatment plans. Additionally, parental input may be influenced by their own anxiety or stress, potentially introducing bias into the diagnostic process. Conversely, as noted in statements such as “It is sometimes difficult to conduct physical examinations such as palpation,” physicians may find it challenging to assess the child without parental input. Wang et al. and Opipari-Arrigan et al. have utilized mobile health technologies to support real-time data sharing and patient-clinician collaboration for the optimal management of chronic pediatric conditions [25,26]. This approach allows parents to record their child’s symptoms and daily life data necessary for medical consultations and share this information with healthcare providers, thereby contributing to improved quality of care. These technologies could also be applied to SM, potentially reducing the burden on parents of children with SM when seeking medical care for their child. Developing a standardized framework for supporting children with SM in collaboration with healthcare providers, along with creating an app that allows parents of children with SM to assess their child’s condition according to these standards, would be crucial steps. Given the well-documented importance of multidisciplinary collaboration in mental health treatment [27], it is essential in SM treatment to involve a team of professionals, such as psychologists, social workers, and nurses, who can collaboratively assess the child’s condition from multiple perspectives. This approach may also alleviate the burden on parents who might otherwise feel solely responsible for managing their child’s diagnosis and treatment, as indicated by segments such as “I must handle everything.”

The second-level category “Healthcare Providers’ Responses” included first-level categories such as “Lack of Understanding of SM by Physicians” and “Cold attitude from healthcare providers.” Previous research [12] similarly indicates that some physicians have made dismissive remarks about SM or appeared uninterested in the child’s SM during examinations. When healthcare providers lack understanding of SM, it exacerbates the difficulties faced by parents. To alleviate these challenges, there is a need to develop training programs on SM for healthcare professionals. By deepening their understanding of SM and teaching them how to appropriately support children with SM and their families, a more reassuring environment for medical visits can be created. Additionally, as this study also highlighted issues at hospital reception, as indicated by segments such as “It is difficult to make the situation understood at the reception desk,” implementing an educational program of SM for the entire medical team would be effective in ensuring comprehensive care for families of children with SM.

This study’s findings highlight the broader implications for clinical practice, suggesting that current healthcare systems may not be adequately equipped to meet the needs of these families.

Building on the findings of this study, future research should focus on several key areas to enhance support for children with SM and their families. First, more in-depth studies are needed to evaluate the long-term effectiveness of telemedicine in reducing anxiety and improving access to healthcare for SM patients. This could involve randomized controlled trials comparing telemedicine with in-person visits to assess outcomes such as anxiety reduction, treatment adherence, and parent satisfaction. Second, the development and testing of psychological preparation protocols tailored specifically for children with SM should be prioritized. Future research could explore how different preparation tools, such as virtual tours of medical facilities or step-by-step visual aids, impact anxiety levels and cooperation during medical visits. Comparative studies could investigate which methods work best for different age groups or severity levels of SM. Third, further investigation is required to better understand how communication tools (e.g., communication cards, mobile health applications) can facilitate interaction between SM children and healthcare providers. Studies should explore the practicality and efficacy of these tools in clinical settings, examining their impact on diagnostic accuracy and reducing the communication burden on parents.

Finally, future research should focus on developing comprehensive training programs for healthcare providers to improve their understanding of SM and parent’s challenges. Additionally, research should explore how these programs affect parental satisfaction and healthcare outcomes, with the goal of creating standardized training modules that can be implemented across various medical specialties.

By adopting these strategies, healthcare systems can create a more supportive and accessible environment for children with SM, helping to alleviate the burden on both the children and their families while improving healthcare outcomes.

This study offers several strengths. First, the use of a qualitative approach, specifically the KJ method, allowed for a deep exploration of the lived experiences of parents of children with SM, capturing nuanced insights into the challenges they face when accessing healthcare services. Additionally, the study’s focus on parental perspectives fills a crucial gap in existing literature, providing a more comprehensive understanding of the barriers in medical care for children with SM. By identifying key areas such as the difficulty of communication during medical visits and the lack of healthcare provider understanding, this study has practical implications for improving healthcare delivery for this population.

However, there are several limitations to consider. The first limitation is the variability in the information regarding the medical departments where parents of children with SM experience challenges when accessing healthcare services. In this study, pediatrics and dentistry were frequently reported as departments where children with SM and their parents encountered significant challenges. Given that pediatric and dental visits are more common among children compared to other specialties [28,29], parents of children with SM are more likely to encounter challenges in these settings. Additionally, the fact that children are more likely to engage in communication during consultations in pediatrics and dentistry, compared to fields such as dermatology or orthopedics, may contribute to the heightened sense of difficulty reported by parents.

The second limitation of this study is the insufficient consideration of the impact that the severity of SM and the presence of comorbidities may have on parental challenges. Specifically, parents may encounter increased challenges when their child has severe SM, while milder cases may result in relatively smoother healthcare visits. Furthermore, Of the children in this study, 22 had comorbid SM and ASD. The second-level category “Influence of Hospital Atmosphere,” as highlighted in the results, includes challenges such as “My child panics at a clinic for the first time.” This difficulty may be attributed not only to SM but also to the presence of comorbid ASD. It is essential to conduct further replication studies to examine how these factors influence parents’ experiences in healthcare settings. Failure to account for these factors may result in a one-dimensional understanding of parental experiences, potentially leading to underestimation or overestimation of the challenges faced by specific groups of parents.

The third limitation of this study is that the participants were recruited through the internet, which may have biased the sample toward parents who have access to the internet or are more inclined to use it. Consequently, the perspectives of parents without internet access or those less willing to use online resources may not have been adequately represented. Additionally, the participants were limited to mothers residing in Japan, and cultural and societal factors may have significantly influenced their experiences. While some of the reported challenges are consistent with previous research, the experiences of parents of children with SM in other countries, or within different healthcare systems and cultural contexts, may differ. Therefore, caution is needed when applying these findings to other nations or cultures.

The fourth limitation is the potential influence of researcher subjectivity when qualitatively analyzing the open-ended data. Although we used the KJ method to systematically organize the data and made efforts to maintain objectivity by incorporating perspectives from multiple researchers, there remains a possibility that researcher bias may have influenced the interpretation. Additionally, the sample size was determined by the number of participants who agreed to take part during the recruitment period, which may limit the transferability of the findings.

Future studies should continue to investigate which medical departments present the greatest challenges for parents of children with SM and identify the specialties where SM-specific training programs are most urgently needed. It should also rigorously assess SM severity and the presence of comorbidities to clarify their impact on parents’ healthcare experiences. Additionally, expanding the sample size to include not only mothers but also other family members and families from diverse countries will facilitate a more comprehensive exploration of relevant themes. To ensure that no critical insights are overlooked, future studies should aim to achieve theoretical saturation.

## Conclusions

Based on the results of this study, it was revealed that parents of children with SM face three primary challenges when accessing healthcare services: “Journey to Receiving Treatment,” “Physicians’ Inability to Accurately Capture the Child’s Condition,” and “Healthcare Providers’ Responses.” To address these challenges faced by parents of children with SM, the utilization of telemedicine and mobile health technologies, multidisciplinary collaboration, and the development of training programs on SM for healthcare professionals were identified as potential strategies for alleviation. In particular, telemedicine and mobile health technologies provide a means of delivering high-quality healthcare services to children with SM and their parents, transcending time and geographical constraints. Therefore, it will become increasingly essential to develop multifaceted approaches to alleviate the challenges faced by parents of children with SM.

## Appendices

**Table 2:** Demographic characteristics and reported challenges in seeking medical care for children with selective mutism. ASD: autism spectrum disorder; ADHD: attention-deficit/hyperactivity disorder; ID: intellectual disability; SepAD: separation anxiety disorder; SM: selective mutism; SocAD: social anxiety disorder

ID	Age of parents	Sex of parents	Age of children	Gender of children	Children’s age at SM diagnosis	Children’s comorbid diagnoses	Medical departments where difficulties were experienced	Challenges encountered during medical visits of parents
1	38	Female	8	Female	4	ASD	Otolaryngology	My child is unable to respond, regardless of what the doctor asks. Doctors frequently ask me, “Is your child autistic?” The doctor often laughs and asks, “Is your child autistic?”
2	41	Female	7	Female	5	ASD	Otolaryngology	Doctors who fail to understand selective mutism often appear annoyed
3	39	Female	6	Female	4	ASD	Pediatrics	My child is often asked by the doctor to say “ah” for a throat examination, but she cannot do it. When with an unfamiliar doctor, my child cannot even speak close to my ear
4	38	Female	9	Female	9	ASD, ADHD	Pediatrics, Dentistry, Orthopedics	My child cannot even enter the examination room. Unable to sit in the chair. My child does not speak. My child does not react
5	43	Female	7	Male	6	ASD	Pediatrics	When my child doesn’t feel comfortable with a new doctor, they tear up and ask to go home
6	35	Female	6	Female	5	ASD, SocAD	Dermatology	My child is unable to answer the doctor’s questions
7	43	Female	11	Male	4	ASD, ID	Pediatrics, Dentistry	My child remains silent no matter what is asked. When asked something, my child freezes
8	43	Female	11	Female	8	ASD	Dentistry, Ophthalmology	When my child didn’t respond to questions from medical staff, they appeared puzzled
9	45	Female	6	Female	6		Dentistry, Dermatology	My child has an aversion to the hospital itself
10	37	Female	12	Male	8	ASD, ADHD, SocAD, SepAD	Pediatrics, Psychiatry	The waiting time is too long
11	39	Female	10	Male	10		Pediatric Psychiatry	Since my child does not respond to the physician’s questions, it becomes impossible to conduct the examination. The doctor can only diagnose my child with “selective mutism.”
12	48	Female	11	Male	7	ASD	Pediatrics, Ophthalmology, Dentistry, Otolaryngology	My child is unable to answer on his own. It takes time for my child to get used to the hospital. There are times when physical examinations, such as palpation, are difficult
13	32	Female	5	Female	4	ASD, ADHD	Pediatrics, Dentistry, Otolaryngology	Unable to answer the doctor’s questions. In cases related to developmental disabilities, my child is often mistaken for having an intellectual disability. Even with the regular doctor, my child sometimes gets scared and tries to run away. My child panics at a clinic for the first time
14	46	Female	11	Female	3		Pediatrics, Dentistry, Internal Medicine	Unless the hospital is open, bright, and clean, it’s impossible. My child can’t go to the hospital if the doctor there isn’t kind
15	34	Female	4	Female	3	ASD, SocAD	Pediatric Sleep Clinic	It’s hard for me to discuss my concerns or negative issues about my child in front of the doctor when my child is sitting next to me
16	48	Female	10	Female	6	ASD	Pediatrics, Dentistry, Ophthalmology	When the doctor spoke to my child and they didn’t respond, it seemed to cause annoyance. My child freezes out of discomfort
17	47	Female	7	Female	6	ASD	Pediatrics, Dentistry, Otolaryngology, Dermatology, Ophthalmology	Unable to follow instructions. Unable to open their mouth. My child is unable to answer the doctor’s questions. My child cannot follow instructions. My child cannot open her mouth. It is often difficult to conduct tests. Since my child finds it difficult to meet new people, medical appointments are challenging
18	44	Female	11	Female	5	ASD	Pediatrics, Dentistry, Ophthalmology	My child is unable to respond verbally. I have difficulty with my child being sensitive to pain and becoming violent
19	47	Female	9	Female	6	ASD	Ophthalmology	Even when the doctor asks questions, my child does not respond. My child freezes. I must handle everything. I feel stressed about explaining to the hospital staff that my child has selective mutism. I have also been treated coldly
20	46	Female	16	Female	13		Internal Medicine, Pediatric Psychiatry	Because my child is looking down, the staff pay unnecessary attention. The face recognition device at the hospital entrance, which automatically measures body temperature, does not respond because my child is looking down
21	37	Female	11	Female	7		Pediatrics, Pediatric Psychiatry, Dermatology	It’s difficult to attend medical appointments because my child cannot leave the house
22	47	Female	15	Male	5		Psychiatry	It’s difficult to schedule appointments consistently. There are long intervals between medical visits
23	44	Female	7	Female	4	ASD	Internal Medicine, Otolaryngology	My child is unable to speak with the doctor. I always have to act as my child’s interpreter
24	35	Female	10	Female	10		Pediatrics, Dentistry, Ophthalmology	My child freezes and is unable to answer the doctor’s questions. There are times when she is unable to effectively communicate her symptoms. My child is unable to respond during vision tests
25	45	Female	16	Female	5	ASD, ID	Dentistry	My child doesn’t speak at all. At every medical appointment, the parent always speaks on the child’s behalf. It takes time for my child to get used to a hospital, so we avoid changing hospitals frequently. Until the early years of elementary school, due to anxiety and nervousness, my child was unable to sit alone in the dentist’s chair
26	39	Female	6	Female	6		Pediatrics, Dentistry	Even with ample preparation, convincing my child takes time, causing us to be late for the scheduled hospital appointment. It is difficult to make the situation understood at the reception desk. I find it exhausting to explain to my child at home about attending the hospital to ensure the medical examination proceeds smoothly. I get exhausted practicing with my child at home to ensure the doctor’s examination proceeds smoothly. Anxiety over dental extractions has made it impossible for my child to leave the house. My child is afraid of getting injections at the hospital
27	46	Female	5	Female	4	ASD	Pediatrics, Dentistry	It’s challenging to plan and support medical visits that involve procedures the child finds difficult, and they rarely succeed
28	44	Female	10	Male	9	ASD	Pediatrics, Dentistry	My child cannot behave in a manner appropriate for his age. He is also unable to respond appropriately for his age
29	42	Female	6	Female	4		Ophthalmology	Unable to respond verbally. My child is unable to perform to their full potential during tests
30	46	Female	10	Male	5	ASD	Pediatrics, Dentistry	My child says they don’t want to go to the hospital. Getting my child to the hospital is an enormous challenge. Even when we manage to go to the hospital, my child does not speak. I was able to take my child to the hospital in the early years of elementary school, but now it’s impossible, and we can no longer go
31	37	Female	5	Female	4		Dentistry, Otolaryngology, Ophthalmology	My child cries when the doctor is overbearing. My child refuses to attend medical appointments if the doctor is male
